# Altered cortical and subcortical morphometric features and asymmetries in the subjective cognitive decline and mild cognitive impairment

**DOI:** 10.3389/fneur.2023.1297028

**Published:** 2023-12-01

**Authors:** Jin Yang, Lingyan Liang, Yichen Wei, Ying Liu, Xiaocheng Li, Jiazhu Huang, Zhiguo Zhang, Linling Li, Demao Deng

**Affiliations:** ^1^School of Medicine, Guangxi University, Nanning, Guangxi, China; ^2^Department of Radiology, The People's Hospital of Guangxi Zhuang Autonomous Region, Guangxi Academy of Medical Science, Nanning, Guangxi, China; ^3^School of Computer Science and Technology, Harbin Institute of Technology, Shenzhen, Guangdong, China; ^4^Marshall Laboratory of Biomedical Engineering, Shenzhen University, Shenzhen, Guangdong, China; ^5^Peng Cheng Laboratory, Shenzhen, Guangdong, China; ^6^School of Biomedical Engineering, Shenzhen University Medical School, Shenzhen University, Shenzhen, China; ^7^Guangdong Provincial Key Laboratory of Biomedical Measurements and Ultrasound Imaging, Shenzhen, China

**Keywords:** subjective cognitive decline, mild cognitive impairment, asymmetry index, local gyrification index, hypothalamic subfields

## Abstract

**Introduction:**

This study aimed to evaluate morphological changes in cortical and subcortical regions and their asymmetrical differences in individuals with subjective cognitive decline (SCD) and mild cognitive impairment (MCI). These morphological changes may provide valuable insights into the early diagnosis and treatment of Alzheimer's disease (AD).

**Methods:**

We conducted structural MRI scans on a cohort comprising 62 SCD patients, 97 MCI patients, and 70 age-, sex-, and years of education-matched healthy controls (HC). Using Freesurfer, we quantified surface area, thickness, the local gyrification index (LGI) of cortical regions, and the volume of subcortical nuclei. Asymmetry measures were also calculated. Additionally, we explored the correlation between morphological changes and clinical variables related to cognitive decline.

**Results:**

Compared to HC, patients with MCI exhibited predominantly left-sided surface morphological changes in various brain regions, including the transverse temporal gyrus, superior temporal gyrus, insula, and pars opercularis. SCD patients showed relatively minor surface morphological changes, primarily in the insula and pars triangularis. Furthermore, MCI patients demonstrated reduced volumes in the anterior-superior region of the right hypothalamus, the fimbria of the bilateral hippocampus, and the anterior region of the left thalamus. These observed morphological changes were significantly associated with clinical ratings of cognitive decline.

**Conclusion:**

The findings of this study suggest that cortical and subcortical morphometric changes may contribute to cognitive impairment in MCI, while compensatory mechanisms may be at play in SCD to preserve cognitive function. These insights have the potential to aid in the early diagnosis and treatment of AD.

## 1 Introduction

Alzheimer's disease (AD) is a neurodegenerative disorder recognized as the most prevalent cause of dementia. It manifests as gradually worsening cognitive dysfunction and behavioral impairment. Patients with AD commonly undergo the stages of subjective cognitive decline (SCD), mild cognitive impairment (MCI), and eventually progress into dementia. MCI is a critical transitional phase between normal aging and AD (1). SCD is characterized by individuals who decline in cognitive capacity but perform normally on neuropsychological tests (2) and may be a clinical condition that precedes MCI in the AD continuum (3, 4). A study suggests an increased risk of SCD progression to MCI or AD (5). Currently, there is no effective treatment or medication for AD, which makes early diagnosis and intervention to preserve cognitive function critical in combating AD (6). Therefore, it is essential to investigate the biomarkers of AD to make early diagnosis and provide better opportunities for early therapy.

Studies have illustrated that brain atrophy severity correlates with AD progression (7). A wealth of research supports that discernable structural brain changes captured through structural magnetic resonance imaging may act as potential indicators of neurodegenerative damage and disease progression in AD patients (8, 9). Structural modifications have been identified in various brain regions of individuals with SCD and MCI, enveloping both cortical and subcortical structures (10, 11). The left-right asymmetry in structure and function is a primary feature of the human brain, and alterations in this asymmetry have been observed in AD (12). In particular, as the disease progresses in AD patients, alterations in the asymmetry of specific brain structures between the left and right hemispheres have been identified (13). The structural brain asymmetry may be a potential biomarker for predicting the early stages of AD (14). Many studies have uncovered asymmetrical abnormalities in the cerebral hemispheres of patients with MCI, primarily relating to cortical thickness, surface area, and the hippocampus (15–17). Regarding the asymmetry of subcortical structure, Z. Fu et al. found significant changes in SCD, MCI, and AD (12). Alterations in hippocampal asymmetry and its sub-regions have been observed in AD patients, suggesting an area of interest for ongoing research. A meta-analysis has illuminated that hippocampal asymmetry exhibits dynamic modifications during the pathological progression from HC, MCI, to AD (18). However, in contrast to the entire hippocampal region, hippocampal sub-regions have demonstrated superior efficacy in identifying AD symptoms (19). Recent investigation suggests that hippocampal sub-region asymmetry may serve as a viable biomarker for assessing MCI and AD (17).

The anterior thalamic nucleus and hypothalamus are crucial components of the Papez circuit and play crucial roles in neurophysiological functions and the pathophysiology of neurodegenerative diseases. Previous studies have documented a notable reduction in overall thalamic volume in MCI and AD patients (20, 21). Furthermore, the anterior and mediodorsal nuclei of the thalamus have been demonstrated to play a significant role in memory functions (22, 23). Hypothalamic atrophy has been highlighted as an early manifestation of AD, deteriorating similarly to the hippocampus as the disease progresses (24). There is a dearth of studies examining asymmetric changes in thalamic and hypothalamic sub-regions, as well as the comprehensive information of the asymmetric changes in cortical and subcortical regions in SCD and MCI.

In this study, we aimed to explore the cortical and subcortical morphometric features and asymmetries in SCD and MCI patients. Simultaneously, given the heterogeneity of the SCD sample, we sought to replicate previous studies on cortical morphological indicators and their asymmetries to assess the stability and reliability of these indicators across different populations. We hypothesized that alterations in specific cortical surface and subcortical structures would be detected in SCD and MCI, and would be more extended in MCI. The asymmetries would be found in the pre-dementia state of AD.

## 2 Materials and methods

### 2.1 Participants

We recruited 99 MCI from the First Affiliated Hospital of the Guangxi University of Chinese Medicine, China, and 62 SCD and 70 age-, sex-, years of education-matched healthy controls (HC) from the local Community and Elderly Activity Center of Nanning City between April 2016 and January 2018. Written informed consent was acquired from all participants.

All participants provided written informed consent to participate in the study, which was approved by the Medicine Ethics Committee of the First Affiliated Hospital, Guangxi University of Chinese Medicine. All research procedures were conducted by the Declaration of Helsinki. This study was registered at https://www.chictr.org.cn/, and the Clinical Trial Registration Number was ChiCTR-IPR-16009144.

### 2.2 Inclusion/exclusion criteria

Two neurologists with more than 5 years of clinical experience completed all neuropsychological evaluations. A neuropsychological test battery was employed to screen the subjects, which included Mini-Mental State Examination (MMSE) (25), Montreal Cognitive Assessment (MoCA) (26), Clinical Dementia Rating (CDR) (27), Geriatric Depression Scale (GDepS) (28), and Global Deterioration Scale (GDS). Moreover, there were six neuropsychological tests to analyze subjects' three cognitive domains (memory, language, and attentive/executive functions), which included Auditory Verbal Learning Test (AVLT delayed recall and AVLT-recognized) (29), Animal Fluency Test (AFT) (30), 30-item Boston Naming Test (BNT) (31), and Trail Making Test (STT-A and STT-B) (32).

The inclusion criteria of all participants are (a) age between 55 and 75 years old, (b) right-handed, (c) Chinese Han nationality, and (d) daily living skills and social vocations were not impacted. The exclusion criteria for all participants in this study are (a) other illnesses that were terminal, severe, or unstable; (b) severe hearing or visual disability; (c) dementia, cerebral infarction, or physical/neurological problems that might cause brain dysfunction; (d) medicines that may induce cognitive alterations or organ failure; and (e) MRI-examination contraindications.

The diagnostic criteria of MCI (33) are described as follows: (a) the chief complaint was memory impairment, which another informed person also confirmed; (b) other cognitive functions were relatively intact or just marginally compromised; (c) daily-life abilities were unaffected; (d) the diagnostic criteria of dementia were not satisfied; (e) additional systemic illnesses that can impair brain function were ruled out; and (f) the MMSE score was 24–27, the CDR score was 0.5, and the GDS score was 2–3.

Participants of the SCD group and HC group determined as follows: (a) the MMSE score was >27, the CDR score was 0, and the GDS score was 1; (b) exclude subjects if any of the following conditions were met: abnormalities on two measures in the same cognitive domain, defined as >1 standard deviation (SD); or if each of the three cognitive domains had an impaired score (defined as > 1 SD) (34); and (c) individuals who had complains of a deteriorating memory were regarded as the SCD patients (2), while individuals who passed neuropsychological tests and had no complaints were included in the HC group.

### 2.3 MRI acquisition

All subjects were scanned on a 3.0-T MRI scanner (Magnetom Verio, Siemens Medical, Erlangen, Germany). The structural MRI data were collected in a sagittal orientation using magnetization-prepared rapid-gradient echo (MPRAGE) sequences with the following imaging parameters: TR = 1,900 ms, TE = 2.22 ms, the field of view (FOV) = 250 mm × 250 mm, slice thickness = 1 mm, matrix size = 256 × 256, flip angle of 9 degrees, and the number of slices 176.

### 2.4 MRI analysis

Structural images were analyzed using the “recon-all” pipeline implemented in the FreeSurfer software (version 7.2.0). For each subject, volume segmentation and cortical surface reconstruction were performed. This study extracted surface area, mean thickness, and local gyrification index (LGI) for each of the 68 cortical regions (34 per hemisphere) in the Desikan-Killiany parcellation scheme. We visually inspected the pial and white surfaces of each subject's axial, sagittal, and coronal sections so that each participant's segmentation results were individually checked and corrected (35).

**Figure 1:**
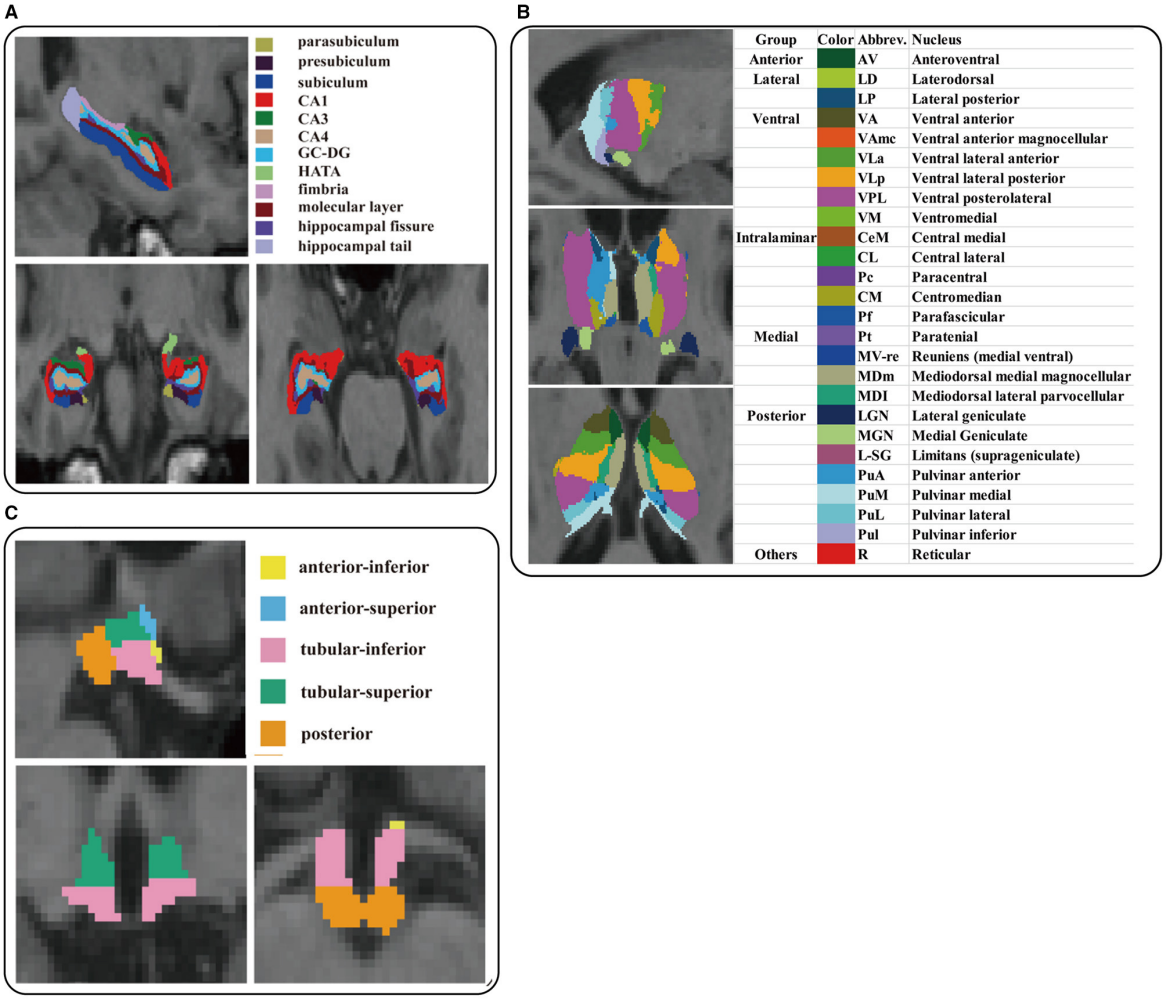
Illustration of anatomical atlases used for the segmentation of subcortical structures: **(A)** hippocampus, **(B)** hypothalamus, and **(C)** thalamus.

Furthermore, we also extracted the volumes of subfields of the hippocampus, hypothalamus, and thalamus because of the importance of considering specific sub-structures instead of considering these structures as a whole (21, 36, 37). The hippocampal segmentation module performed automated hippocampal subregion segmentation by Bayesian inference technique to obtain the 12 hippocampal subregions: parasubiculum, presubiculum, subiculum, CA1, CA3, CA4, granule cells in the molecular layer of the dentate gyrus (GC-ML-DG), hippocampal-amygdaloid transition area (HATA), fimbria, molecular layer, hippocampal fissure, and hippocampal tail (38, 39). The hypothalamic segmentation module is based on deep convolutional neural networks to accurately segment the hypothalamus into the following five sub-nuclei: anterior-inferior, anterior-superior, posterior, tubular-inferior, and tubular-superior (40). The thalamic segmentation module relies on probabilistic mapping of *ex vivo* MRI and histological data to segment the thalamus into 26 sub-regions, which are divided into six main thalamic regions: posterior, medial, intralaminar, ventral, lateral, and anterior nuclei (41). Each subject's estimated total intracranial volume (eTIV) was also collected for further analysis using covariates. After segmentation, all results were visually inspected, and subjects were discharged for quality control if there were segmentation errors. The results of the hippocampal sub-region, hypothalamic sub-region, and thalamic sub-region segmentation are shown in Figure 1.

### 2.5 Asymmetry index

The asymmetry index (AI) was calculated for each cortical and subcortical region to quantity the differences between the left and right hemispheres using the following formula: AI = (left – right) × 100 / (left + right), where left and right represent the corresponding left and right morphometric features (volume of subcortical structures, thickness, surface area, or LGI of cortical regions) (42, 43), and the values of AI range from −100 (complete right-lateralized asymmetry) to +100 (complete left-lateralized asymmetry).

### 2.6 Statistical analysis

Clinical and demographic data were analyzed using SPSS software (IBM Inc., Armonk, New York, USA). Continuous variables were tested for normality (Shapiro-Wilk test). Differences in age, education, MMSE scores, GDepS scores, and MoCA scores among the three groups were determined via analysis of variance (ANOVA) or Kruskal-Wallis tests. Differences in the gender distribution were assessed with a Pearson Chi-square test. ROI-wise analysis of covariance (ANCOVA), adjusted for age, gender, years of education, and eTIV, was used to assess the main effects of the group (HC, SCD, and MCI) on each morphometric feature and AI. *Post-hoc* tests with Bonferroni's correction were used to follow up on significant main effects. A two-tailed *p-*value < 0.05 was considered statistically significant. Furthermore, we investigated the relationship between morphometric features with significant group differences and clinical variables (MMSE, MoCA, GDeps) using Pearson's partial correlation analyses controlled for age, gender, education, and eTIV. Due to the exploratory nature of the analysis, *p*-values were uncorrected for multiple comparisons (44–47).

## 3 Results

### 3.1 Demographic and clinical characteristics

Ninety-seven patients were initially included in the MCI group, and two were excluded due to segmentation errors. Table 1 presents the demographic information and neuropsychological characteristics of the HC, SCD, and MCI groups. There were no significant differences in age and eTIV among the three groups, and Chi-square tests did not reveal any differences in gender distribution. Significant differences were observed in MMSE scores between SCD and MCI, as well as between HC and MCI. MoCA scores manifested significant differences between any two compared groups, and GDepS scores revealed significant differences between HC and MCI.

**Table 1:** Demographic and clinical characteristics of SCD and MCI patients, and healthy controls.

	**HC (*n =* 70)**	**SCD (*n =* 62)**	**MCI (*n =* 97)**	***P* value**	***Post-hoc***		
					**SCD vs. HC**	**MCI vs. HC**	**SCD vs. MCI**
**Age**	64.64 ± 5.76	64.85 ± 5.62	65.18 ± 6.56	0.945	NS	NS	NS
**Gender (men/women)**	26/44	20/42	28/69	0.529	NS	NS	NS
**Education (years)**	11.87 ± 3^b^	11.9 ± 3.02^c^	10.65 ± 2.91^bc^	0.004^*^	1.00	0.017	0.012
eTIV (^*^10^**6**^ **mm**^**3**^**)**	1.41 ± 0.15	1.4 ± 0.15	1.39 ± 0.13	0.859	NS	NS	NS
**MMSE**	29.13 ± 0.74^b^	28.89 ± 0.85^c^	25.85 ± 1.03^bc^	< 0.001^*^	1.00	< 0.001	< 0.001
**MoCA**	26.11 ± 2.01^ab^	24.85 ± 2.49^ac^	21.35 ± 3.03^bc^	< 0.001^*^	0.028	< 0.001	< 0.001
**GDepS**	4.17 ± 2.41^b^	4.74 ± 2.66	5.6 ± 2.03^b^	< 0.001^*^	0.67	0.001	0.088
**CDR**	0	0	0.5	-	-	-	-

HC, healthy control; MCI, mild cognitive impairment; SCD, subjective cognitive decline; NS, not significant. The data showed as mean ± standard deviation. Age, education, MoCA scores, MMSE scores, and GDepS scores were tested via analysis of variance (ANOVA) or Kruskal-Wallis tests. The chi-square was employed to examine the differences in sex distribution.

* represents a significant difference among the three groups (*p* < 0.05), a represents a significant difference between the HC and SCD groups (*P* < 0.05), b represents a significant difference between the HC and MCI groups (*P* < 0.05), and c represents a significant difference between the SCD and MCI groups (*p* < 0.05).

### 3.2 Alterations in morphological measurements

**Figure 2:**
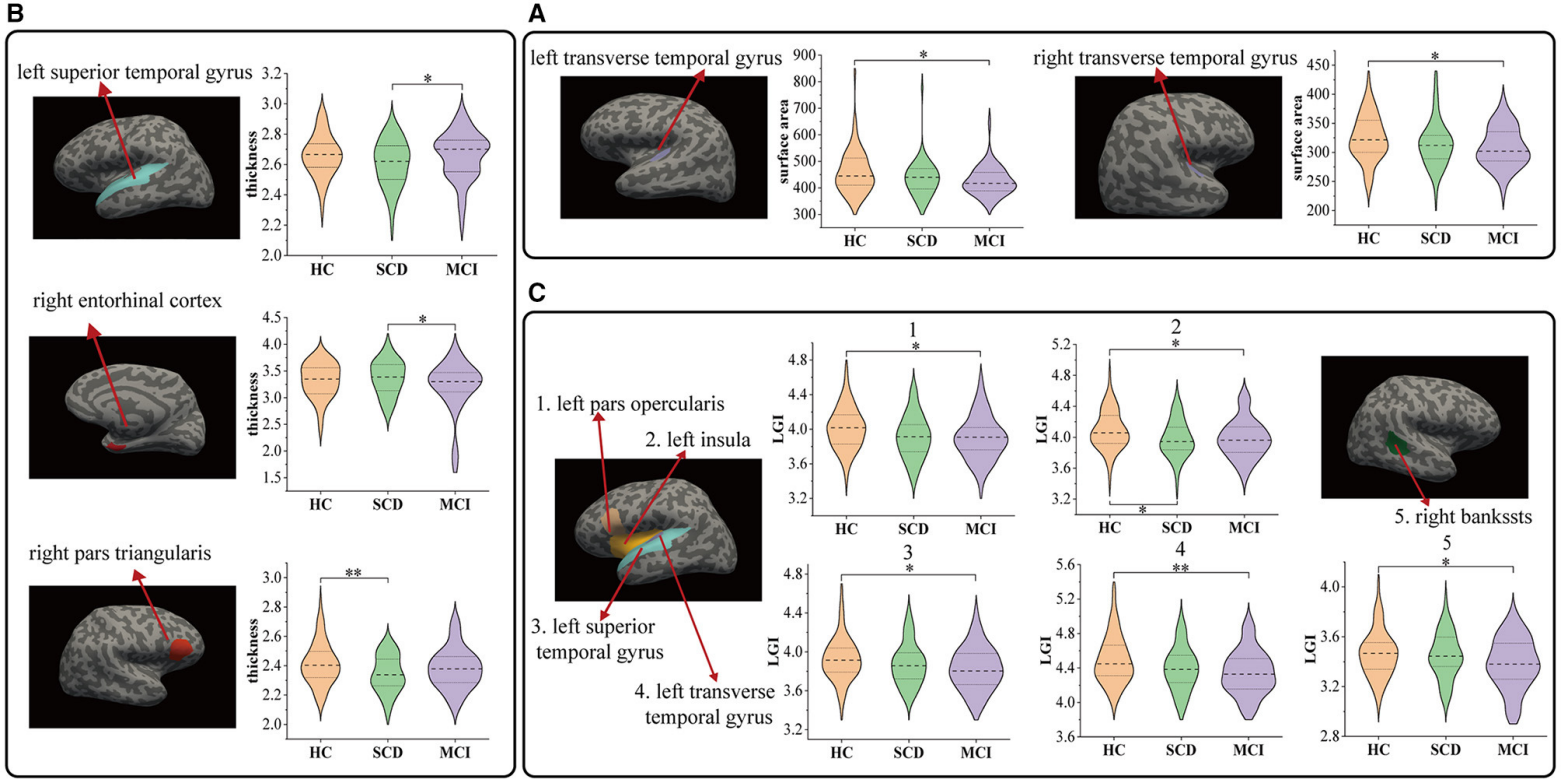
Comparison of cortical region indicators HC and patients with SCD, MCI: **(A)** results of surface area, **(B)** results of thickness, and **(C)** results of local gyrification index. *P < 0.05, **P < 0.01.

Figure 2 and Table 2 depict the significant inter-group differences in morphological features of cortical regions. Specifically, the cortical surface area value of the left transverse temporal gyrus (95% CI = 5.146, 53.411; *p* = 0.011, $\eta_{\text{p}}^{2}$ = 0.04) and the right transverse temporal gyrus significantly diminished (95% CI = 1.773, 27.41; p = 0.02, $\eta_{\text{p}}^{2}$ = 0.033) in MCI compared to HC. Regarding cortical thickness, notable differences were identified in the left superior temporal gyrus (95% CI = −0.111, −0.004; *p* = 0.032, $\eta_{\text{p}}^{2}$ = 0.031) and the right entorhinal cortex (95% CI = 0.002, 0.311; *p* = 0.046, $\eta_{\text{p}}^{2}$ = 0.028) between MCI and SCD and in the right pars triangularis (95% CI = 0.014, 0.128; *p* = 0.009, $\eta_{\text{p}}^{2}$ = 0.039) between SCD and HC. Additionally, compared to HC, MCI exhibited a significant decline in cortical LGI values in the left pars opercularis (95% CI = 0.014, 0.201; *p* = 0.019, $\eta_{\text{p}}^{2}$ = 0.036), left insula (95% CI = 0.011, 0.187; *p* = 0.023, $\eta_{\text{p}}^{2}$ = 0.039), left superior temporal gyrus (95% CI = 0.006, 0.161; *p* = 0.029, $\eta_{\text{p}}^{2}$ = 0.03), left transverse temporal gyrus (95% CI = 0.043, 0.226; *p* = 0.001, $\eta_{\text{p}}^{2}$ = 0.055) and right bankssts (95% CI = 0.0002, 0.138; *p* = 0.049, $\eta_{\text{p}}^{2}$ = 0.031). SCD significantly decreased the left insula (95% CI = 0.003, 0.196; *p* = 0.041, $\eta_{\text{p}}^{2}$ = 0.039).

**Table 2:** Cortical regions with significant inter-group differences in morphological features.

	**Mean** ±**SD**				**Pairwise comparisons** ***p*****-values**		
	**HC**	**SCD**	**MCI**	**ANCOVA**	**SCD vs. HC**	**MCI vs. HC**	**SCD vs. MCI**
**Surface area (mm** ^2^ **)**							
Transverse Temporal Gyrus L	460.46 ± 82.75	434.56 ± 66.53	426.19 ± 55.29	0.011^*^	0.095	0.011^*^	1.00
Transverse Temporal Gyrus R	324.81 ± 39.14	314.13 ± 39.47	308.79 ± 34.52	0.024^*^	0.327	0.02^*^	1.00
**Thickness (mm)**							
Superior Temporal Gyrus L	2.66 ± 0.14	2.61 ± 0.15	2.66 ± 0.16	0.030^*^	0.124	1.00	0.032^*^
Pars Triangularis R	2.41 ± 0.14	2.34 ± 0.13	2.38 ± 0.14	0.012^*^	0.009^*^	0.33	0.317
Entorhinal Cortex R	3.32 ± 0.33	3.37 ± 0.33	3.21 ± 0.47	0.041^*^	1.00	0.289	0.046^*^
**Local gyrification index**							
Pars opercularis L	4.01 ± 0.24	3.92 ± 0.25	3.90 ± 0.25	0.017^*^	0.107	0.019^*^	1.00
Transverse Temporal Gyrus L	4.5 ± 0.25	4.39 ± 0.25	4.35 ± 0.26	0.002^**^	0.074	0.001^**^	0.893
Insula L	4.09 ± 0.25	3.98 ± 0.24	3.98 ± 0.25	0.031^*^	0.041^*^	0.023^*^	1.00
Superior Temporal Gyrus L	3.93 ± 0.22	3.87 ± 0.22	3.83 ± 0.22	0.035^*^	0.635	0.029^*^	0.699
Bankssts R	3.46 ± 0.19	3.45 ± 0.18	3.38 ± 0.20	0.031^*^	1.00	0.049^*^	0.137

HC, healthy control; SCD, subjective cognitive decline; MCI, mild cognitive impairment; R, right; L, left. The data showed as mean ± standard deviation.

* *P* < 0.05,

** *P* < 0.01.

**Figure 3:**
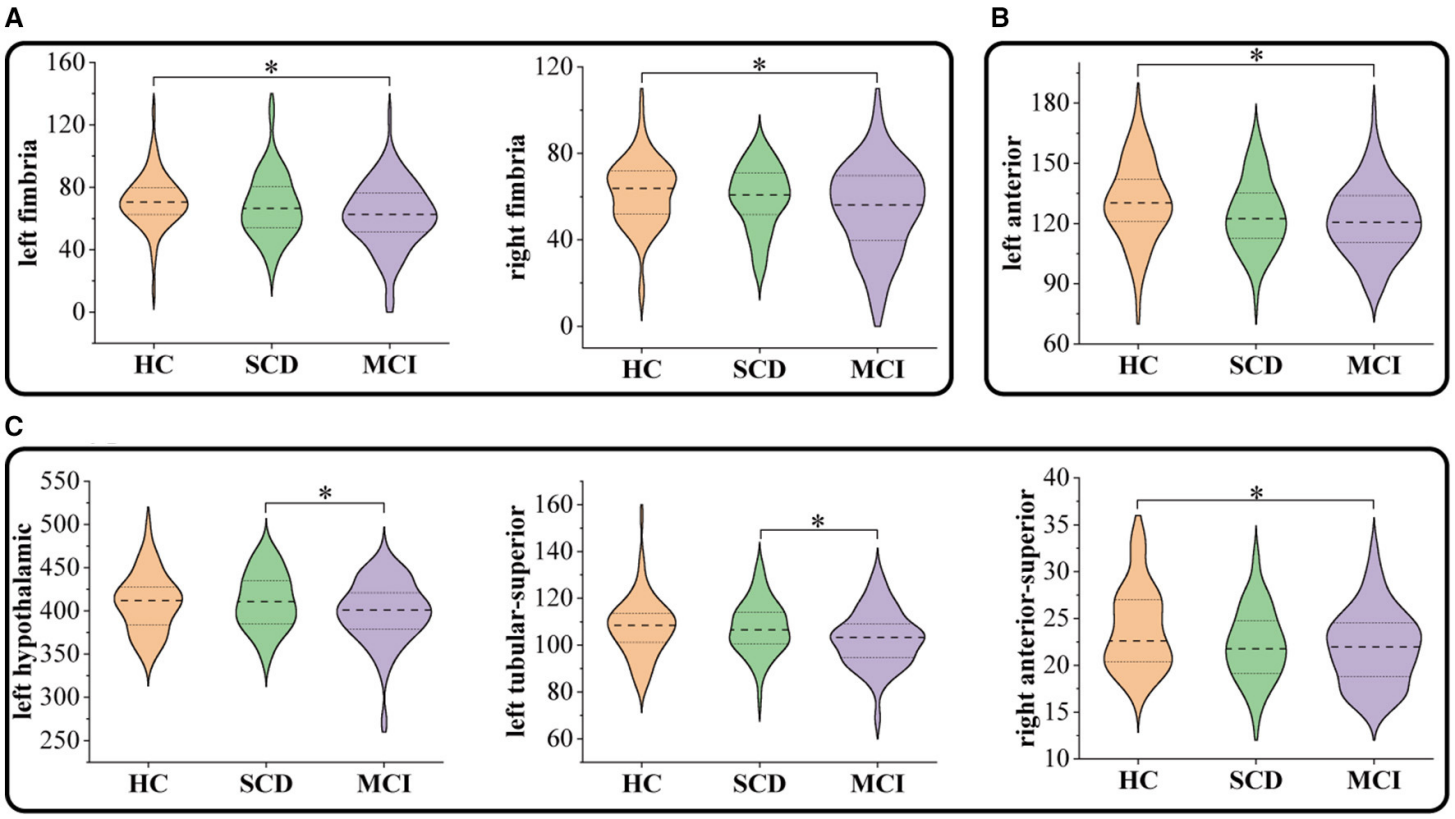
Comparison of subcortical volumes in HC, SCD, and MCI groups: **(A)** hippocampus, **(B)** thalamus, and **(C)** hypothalamus. *P < 0.05.

Figure 3 and Table 3 present the significant inter-group differences observed in subcortical structures. Significant differences were identified in the left fimbria (95% CI = 0.709, 14.46; *p* = 0.025, $\eta_{\text{p}}^{2}$ = 0.033), right fimbria (95% CI = 0.37, 12.26; *p* = 0.033, $\eta_{\text{p}}^{2}$ = 0.031) of the hippocampus, the anterior part of the left thalamus (95% CI = 0.967, 13.82; *p* = 0.018, $\eta_{\text{p}}^{2}$ = 0.035), and the anterior-superior part of the right hypothalamus (95% CI = 0.265, 3.25; *p* = 0.015, $\eta_{\text{p}}^{2}$ = 0.039) between MCI and HC. Compared to SCD, the MCI group exhibited a significant reduction in the volume of the left hypothalamus (95% CI = 1.26, 21.944; *p* = 0.022, $\eta_{\text{p}}^{2}$ = 0.033), and its tubular-superior part (95% CI = 0.292, 8.292; *p* = 0.031, $\eta_{\text{p}}^{2}$ = 0.038). No significant difference was observed between SCD and HC.

**Table 3:** The area of subcortical structures with significant inter-group differences in morphological features.

	**Mean** ±**SD**				**Pairwise comparisons** ***P*****-values**		
	**HC**	**SCD**	**MCI**	**ANCOVA**	**SCD vs. HC**	**MCI vs. HC**	**SCD vs. MCI**
**Hippocampus**							
Fimbria L	71.14 ± 16.9	68.15 ± 20.43	62.04 ± 20.99	0.024^*^	1.00	0.025^*^	0.236
Fimbria R	61.76 ± 15.44	59.51 ± 14.62	54.39 ± 20.95	0.029^*^	1.00	0.033^*^	0.224
**Thalamus**							
Anterior L	130.83 ± 20.11	124.26 ± 17.52	121.9 ± 17.76	0.019^*^	0.147	0.018^*^	1.00
**Hypothalamus**							
Hypothalamus L	409.78 ± 34.28	411.77 ± 32.77	398.83 ± 36.04	0.023^*^	0.935	0.278	0.022^*^
Tubular-Superior L	107.66 ± 12.71	107.4 ± 11.24	102.91 ± 12.1	0.014^*^	1.00	0.057	0.031^*^
Anterior-Superior R	23.9 ± 4.43	22.19 ± 3.82	21.94 ± 4	0.012^*^	0.062	0.015^*^	1.00

HC, healthy control; SCD, subjective cognitive decline; MCI, mild cognitive impairment; R, right; L, left. The data showed as mean ± standard deviation.

* *P* < 0.05.

### 3.3 Alterations in morphological asymmetries

**Figure 4:**
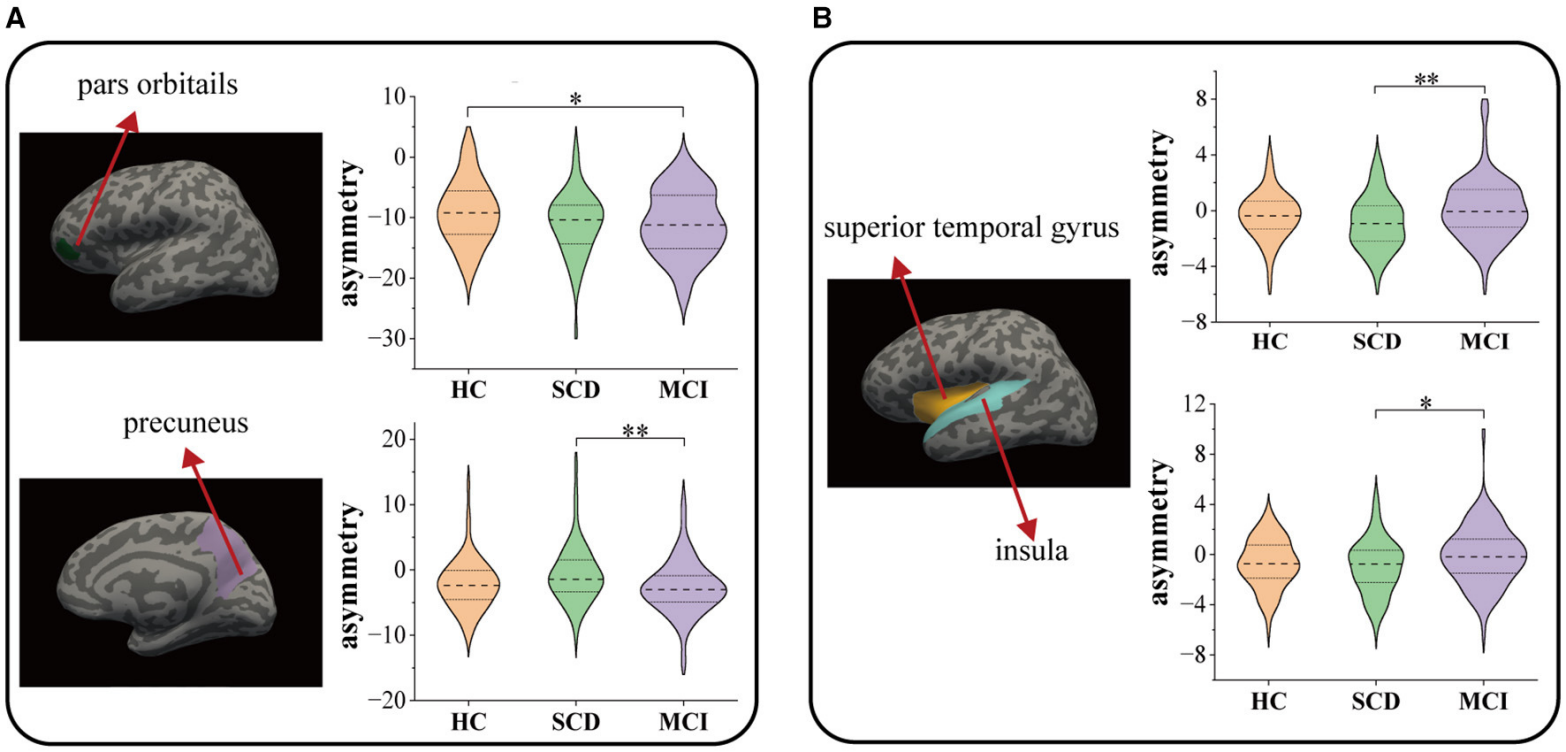
Comparison among HC, SCD, and MCI on the morphological asymmetries: **(A)** surface area and **(B)** thickness. * *P* < 0.05, ** *P* < 0.01.

Figure 4 and Table 4 summarize the significant inter-group differences in morphological feature asymmetry observed in cortical regions. Surface area asymmetry in the pars orbitalis (95% CI = 0.009, 4.086; *p* = 0.049, $\eta_{\text{p}}^{2}$ = 0.03) between HC and MCI, and in the precuneus (95% CI = 0.427, 3.778; *p* = 0.008, $\eta_{\text{p}}^{2}$ = 0.041) between SCD and MCI revealed significant differences. Moreover, significant differences in cortical thickness asymmetry were noted in the superior temporal gyrus (95% CI = −1.776, −0.2; *p* = 0.008, $\eta_{\text{p}}^{2}$ = 0.04) and insula (95% CI = −1.845, −0.125; *p* = 0.019, $\eta_{\text{p}}^{2}$ = 0.038) between SCD and MCI. The inter-group ANCOVA with Bonferroni correction did not unveil any significant differences in the AI values of subcortical structures.

**Table 4:** Cortical regions with significant inter-group differences in asymmetry.

	**Mean** ±**SD**				**Pairwise comparisons** ***P*****-values**		
	**HC**	**SCD**	**MCI**	**ANCOVA**	**SCD vs. HC**	**MCI vs. HC**	**SCD vs. MCI**
**Surface area**							
Pars Orbitalis	−9.09 ± 5.25	−11.01 ± 5.21	−10.98 ± 5.4	0.033^*^	0.101	0.049^*^	1.00
Precuneus	−2.21 ± 3.92	−0.61 ± 4.47	−2.66 ± 4.15	0.009^**^	0.087	1.00	0.008^**^
**Thickness**							
Superior temporal gyrus	−0.37 ± 1.69	−0.86 ± 1.9	0.13 ± 2.19	0.011^*^	0.414	0.405	0.008^**^
Insula	−0.71 ± 1.93	−0.97 ± 2.13	−0.1 ± 2.35	0.014^*^	1.00	0.112	0.019^*^

HC, healthy control; SCD, subjective cognitive decline; MCI, mild cognitive impairment. The data showed as mean ± standard deviation.

* P < 0.05;

** *P* < 0.01.

### 3.4 Correlation with clinical variables

**Figure 5:**
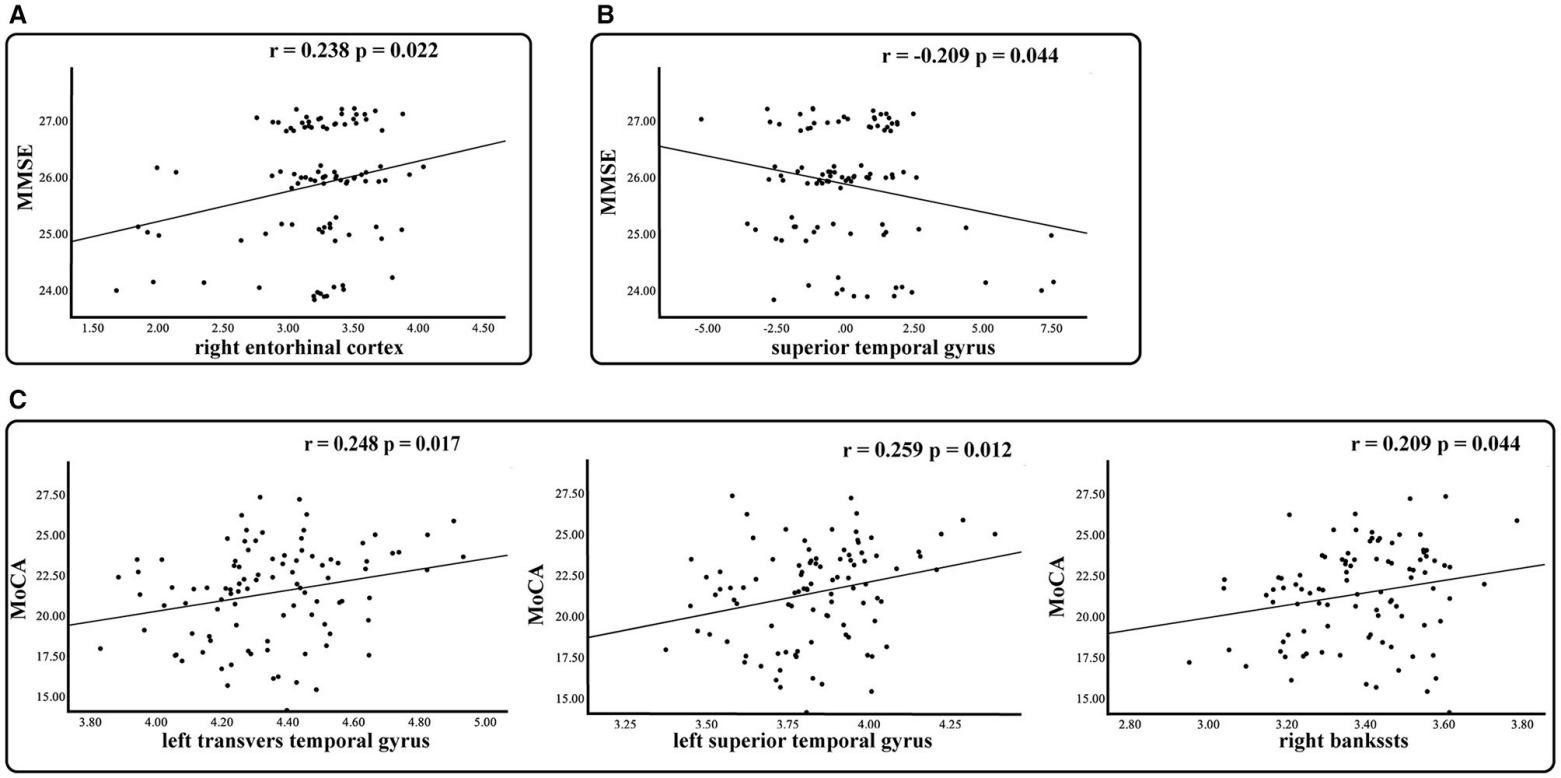
Relationship between morphological measures with significant group differences and clinical measures in the MCI group. Partial *r* and *p* values were obtained after adjustment for age, gender, education, and eTIV. **(A)** Thickness. **(B)** Asymmetry of thickness. **(C)** Local gyrification index.

Within the MCI group, a substantial relationship was discovered between MMSE scores and both the thickness of the right entorhinal cortex (r = 0.238, *p* = 0.022) and the thickness asymmetry of the superior temporal gyrus (r = −0.209, *p* = 0.044). Additionally, MoCA scores exhibited a significant correlation with the LGI of the left transverse temporal gyrus (r = 0.248, *p* = 0.017), the left superior temporal gyrus (r = 0.259, *p* = 0.012), and the right bankssts (r = 0.209, *p* = 0.044) (Figure 5). No significant correlation was observed for the SCD group.

## 4 Discussion

In this cross-sectional study, we investigated the cortical and subcortical morphometric features and asymmetries in SCD and MCI, particularly the asymmetry of thalamic and hypothalamic sub-regions. We believe that the alterations in the thalamus and hypothalamus hold substantial practical significance and value for a more comprehensive understanding of AD, thus furnishing a more precise scientific foundation for the disease's prevention and early intervention strategies. There are three principal findings. First, compared to the HC group, we found reduced cortical LGI values in SCD (the left insula) and MCI (the left insula, the left pars opercularis, left transverse temporal gyrus, left superior temporal gyrus, and right bankssts). Second, regarding the hypothalamus and its sub-regions in MCI, our study demonstrated reduced volume of the anterior-superior region of the right hypothalamus compared to HC and decreased volume of the tubular-superior part of the left hypothalamus compared to SCD. Finally, our study showed significant differences in cortical asymmetry in the pars orbitalis, superior temporal gyrus, and insula in MCI compared to HC and in precuneus compared to SCD. We confirmed some previously reported observations and added new manifestations of cerebral changes in SCD and MCI. Our study suggests that MCI patients show predominantly left-sided atrophy in multiple brain regions compared to HC, while SCD patients exhibit relatively little atrophy. Furthermore, our findings were strengthened by the significant relationships between the observed aberrant morphological measurements and the clinical ratings of cognitive decline.

### 4.1 Cortical morphological alteration

Compared to HC, morphological alterations were observed in the right pars triangularis and left insula in SCD. Previous studies have documented insular atrophy in both SCD and MCI patients (48, 49), suggesting that changes in this region could serve as a reliable neuroanatomical marker for early AD, given the direct or indirect role of the insular cortex in memory processes (50). Our findings suggested that cerebral alterations might precede behavioral cognitive decline in SCD subjects. Consistent with previous literature (51), we observed a significant decrease in cortical surface area of the bilateral transverse temporal gyrus in MCI patients. Interestingly, we observed a trend of reduction in the cortical surface area of these brain regions in SCD, indicating the involvement of bilateral transverse temporal gyrus in preclinical AD and development with the disease progression.

Compared to SCD, MCI patients showed increased cortical thickness in the left superior temporal gyrus, and its asymmetry values had a significant negative correlation with MMSE scores. In addition, the superior temporal gyrus showed rightward asymmetry in HC and SCD and leftward asymmetry in MCI, and this change of laterality may inhibit the coordination of brain function and thus lead to cognitive decline. The entorhinal cortex, located in the medial temporal lobe, is an early site of neurodegeneration in AD. Previous studies have suggested that alterations in the thickness of the entorhinal cortex may act as a reliable biomarker for diagnosing MCI (52). Our results indicated a trend of cortical thinning in this region in MCI and a trend of cortical enlargement in SCD. These findings suggested that the cortical thinning of the entorhinal cortex in MCI was related to cognitive impairment, while there was compensation to retain the cognitive function in SCD. The precuneus functionally involves visuospatial imagery, episodic memory retrieval, self-processing, and consciousness (53). The precuneus's cortical thinning or gray matter atrophy has been reported in MCI (54) and SCD (55). In the present study, the precuneus in MCI patients showed an asymmetric increase in surface area to the right compared to SCD, similar to previous studies. This observed phenomenon may be attributed to the precuneus's heightened vulnerability to neurodegenerative processes in the early stages of AD. Studies indicated more significant metabolic dysfunction in the left hemisphere compared to the right hemisphere in AD patients (56), resulting in the left hemisphere of the precuneus being more prone to degeneration than the right hemisphere.

Our study revealed significant morphological differences in MCI as measured by LGI. The LGI of cerebral regions can quantify the extent of cortical folding, and abnormal changes in cortical folding are associated with cognitive function in older individuals (57). We also observed a significant decrease in LGI in the left hemisphere (the pars opercularis, transverse temporal gyrus, superior temporal gyrus, insula) and right hemisphere (the bankssts). In addition, the LGI values in the left transverse temporal gyrus, left superior temporal gyrus, and right bankssts in the MCI group were positively correlated with the MoCA scale ratings. These results supported previous finding that patients with early AD exhibit lower overall cortical gyrus than cognitively normal controls (58), confirming AD-like brain atrophy patterns in MCI and its correlation with cognitive decline.

### 4.2 Subcortical morphological alteration

According to existing literatures, many neuroimaging investigations have identified atrophy of the subcortical structure, including the hippocampus, amygdala, and caudate, as a distinctive feature of AD and MCI (59). Volume abnormalities in these regions have also been observed in SCD (46, 60). However, our results did not show significant abnormality in these regions, probably because SCD was recruited from the community who did not seek medical help due to the relatively mild cognitive decline.

Our findings showed a significant reduction in the volume of the bilateral fimbria of the hippocampus in MCI patients. This finding is consistent with previous studies, which showed a significant reduction in fimbria volume in MCI patients (46). The fimbria, part of the hippocampus, plays a crucial role in the input and output pathways of the hippocampus. A previous study has suggested that a smaller fimbria is associated with poorer cognitive function in patients with AD (61). This association may be related to the fimbria's anatomical connections and functional pathways. Consequently, the reduced volume of the fimbria may impact the transmission of information within the hippocampus in MCI, potentially contributing to cognitive decline. Research indicated that hippocampal atrophy is a significant hallmark of AD and that atrophy of hippocampal sub-regions in MCI patients occurs mainly in the CA1 and subiculum (62). However, this was not reflected in the present study, possibly due to differences in segmentation methods (manual or automatic), algorithms, and the heterogeneity among patients.

For MCI patients, significantly decreased volumes were observed in the anterior-superior nucleus of the right hypothalamus compared to HC and in the left hypothalamus and its tubular-superior nucleus compared to SCD, which has not been reported before. Previous research has revealed that in addition to cognitive decline, individuals with AD often exhibit a range of non-cognitive symptoms such as weight loss, sleep disorders, metabolic abnormalities, and circadian rhythm disorders (63). Intriguingly, these non-cognitive symptoms often manifest prior to a significant deterioration in cognitive function and tend to exacerbate as the disease progresses. Additionally, studies have identified a potential link between these non-cognitive symptoms and hypothalamic dysfunction (64, 65). Amyloid plaques and neurofibrillary tangles have been detected in the hypothalamus of AD patients (66), and hypothalamic atrophy could be found at early stages of AD (67). Furthermore, hypothalamic GM density decreased with increasing clinical severity, comparable to the degree of hippocampal degeneration (24). These findings support the hypothesis that hypothalamic atrophy is an early manifestation of AD. The anterior-superior nucleus comprises the preoptic area and paraventricular nucleus (PVN). Notably, the PVN in the anterior-superior region of the hypothalamus regulates the hypothalamic-pituitary-adrenal axis (HPA axis). The HPA axis, a universally recognized endocrine target in various neural systems (68), has been observed to be dysregulated in AD, accelerating disease progression and cognitive decline (69). In addition, this study found that the volumes of the left hypothalamus and the tubular-superior nucleus of the left hypothalamus were significantly smaller in MCI than in SCD. The tubular-superior region includes the dorsomedial nucleus (DMN), the PVN, and the lateral hypothalamus. The DMN is a crucial relay site for the PVN and is closely associated with it (70). Notably, the PVN is involved in regulating the HPA axis, and thus atrophy of the tubular-superior region may impact HPA axis regulation, resulting in cognitive decline.

Thalamic atrophy has been closely linked with cognitive impairments, particularly evident in AD and MCI (21). Our study has revealed a significant reduction in the volume of the anterior thalamic nucleus in MCI patients compared to the HC. The anterior thalamic nucleus is a crucial component of the Papez circuit, which is crucial in cognition such as learning and memory process (71). Previous research has unveiled a connection between damage to the Papez circuit and deficits in episodic memory, and the anterior thalamic nucleus played a pivotal role in cognitive decline in AD patients (23). The observed reduction in the volume of the anterior thalamic nucleus in MCI patients substantiates its potential association with early memory and attention losses in AD.

The human brain is characterized by networks consisting of different brain regions to support complex brain functions. Neurological dysfunction is not the result of the alterations in a single brain region but of the interaction of many brain regions. The results of morphometric features located in different regions in the present study supported the complexity of the brain function and reflect the complexity of the pathological mechanism of AD.

### 4.3 Limitations and future direction

This study has several significant limitations. First, the sample size of this study is relatively small, and no repeated MRI scanning was involved. The reported statistical results were not corrected for multiple comparisons, which might increase the risk of false positives and cause the discovered morphometric features to be scattered across different brain regions. We conducted extra analyses to assess our results' reproducibility (as shown in Appendix A), and the stability of results is better when the sample size is more extensive. Besides, although the structural features in MRI are relatively stable (72), it is still necessary to assess the test-retest reliability of the morphometric indices used in the current study (73, 74). It is recommended to repeat this study with a larger sample size and repeated MRI scanning to confirm the reported results. Second, this study lacks a comprehensive consideration of the relationships between changes occurring in different brain structural areas. Future research could consider exploring structural brain networks to gain a more comprehensive understanding of complex brain abnormalities and their interrelationships in different brain regions in AD. Third, the MCI group in this study had significantly lower educational attainment than the other two groups. It is noteworthy that prior studies have reported that the individual's education is inversely associated with cognitive impairment (75–77). Educational level was used as a covariate in the statistical analyses, but it may not be possible to completely exclude the effect of differences in education attainment on the results. Follow-up studies could attempt to group subjects according to different levels of education. Fourth, AD patients should be included to make a more comprehensive view of brain atrophy in the AD continuum. Furthermore, instead of the cross-sectional design adopted here, longitudinal studies should be conducted to verify the causal links between these observed morphological alterations and AD development.

## 5 Conclusions

In summary, our study revealed morphological changes in cortical structures in SCD and MCI, which were more extensive in MCI, suggesting SCD as an intermediate state between MCI and normal aging. Moreover, morphometric features of subcortical structures were detected in MCI. However, they were not significantly found in SCD, indicating that the morphological changes of cortical precede that of subcortical structure in the AD continuum. The current study may contribute to a better understanding of the differences in brain structure in SCD and MCI and provide better biomarkers for effective diagnosis and treatment of early AD.

## Data availability statement

The raw data supporting the conclusions of this article will be made available by the authors, without undue reservation.

## Ethics statement

The studies involving humans were approved by the Medicine Ethics Committee of the First Affiliated Hospital, Guangxi University of Chinese Medicine. The studies were conducted in accordance with the local legislation and institutional requirements. The participants provided their written informed consent to participate in this study.

## Author contributions

JY: Data curation, Formal analysis, Visualization, Writing – original draft, Writing – review & editing. LLia: Visualization, Writing – review & editing. YW: Resources, Visualization, Writing – review & editing. YL: Resources, Writing – review & editing. XL: Resources, Writing – review & editing. JH: Resources, Writing – review & editing. ZZ: Data curation, Validation, Writing – review & editing. LLi: Methodology, Writing – review & editing. DD: Conceptualization, Supervision, Writing – review & editing.
