# Supplementary material for: Altered cortical and subcortical morphometric features and asymmetries in the subjective cognitive decline and mild cognitive impairment
Source: Front Neurol. 2023 Dec 1;14:1297028. doi: 10.3389/fneur.2023.1297028 (PMC10722314; doi:10.3389/fneur.2023.1297028)
Supplement: Supplementary file 1 [file Data_Sheet_1.PDF]

SUPPLEMENTARY MATERIAL

Altered cortical and subcortical morphometric features and asymmetries in the subjective cognitive decline and mild cognitive impairment

Appendix A: the analyses to assess the results' reproducibility

We conducted some additional analysis to check the reproducibility of our results. Specifically, we randomly selected 70%, 80%, and 90% of subjects from each group (SCD, MCI, HC) and repeated the same statistical analyses as performed in the manuscript. This procedure was repeated 100 times and then we calculated the percentage of significant results (P<0.05).

Supplementary Tables

Table S1. Frequency of Maintained Statistical Significance in Brain Regions Across Different Sampling Percentages

	ANCOVA[a]	Sampling Percentage<br>70%<br>ANCOVA[b]	80%<br>ANCOVA[b]	90%<br>ANCOVA[b]
**Surface area**				
Transverse Temporal Gyrus L	P = 0.011	57%	70%	78%
Transverse Temporal Gyrus R	P = 0.024	57%	55%	57%
**Thickness**				
Superior Temporal Gyrus L	P = 0.041	32%	48%	51%
Entorhinal Cortex R	P = 0.012	60%	62%	75%
Pars Triangularis R	P = 0.017	57%	56%	64%
**Local gyrification index**				
Pars Opercularis L	P = 0.017	57%	56%	64%
Superior Temporal Gyrus L	P = 0.035	45%	53%	53%
Transverse Temporal Gyrus L	P = 0.002	77%	80%	90%
Insula L	P = 0.031	55%	55%	68%
Bankssts R	P = 0.031	51%	55%	60%
**Hippocampus**				
Fimbria L	P = 0.024	45%	59%	59%
Fimbria R	P = 0.029	41%	52%	49%
**Hypothalamus**				
Tubular-Superior L	P = 0.014	57%	54%	70%
Hypothalamus L	P = 0.023	49%	54%	57%
Anterior-Superior R	P = 0.012	55%	63%	64%
**Thalamus**				
Anterior L	P = 0.019	50%	56%	68%
**Surface area asymmetry**				

Pars Orbitalis	P = 0.033	45%	37%	52%
Precuneus	P = 0.009	56%	66%	71%
**Thickness asymmetry**				
Superior Temporal Gyrus	P = 0.011	63%	63%	71%
Insula	P = 0.014	60%	63%	68%

a: the results of ANCOVA analysis using the original sample groups.

b: the results of ANCOVA analysis using the randomly selected sample groups, which was calculated as the percentage of significant results. R, right; L, left.
